# A Review of the Challenge-Hindrance Stress Model: Recent Advances, Expanded Paradigms, and Recommendations for Future Research

**DOI:** 10.3389/fpsyg.2020.560346

**Published:** 2020-11-05

**Authors:** Kristin A. Horan, Wheeler H. Nakahara, Michael J. DiStaso, Steve M. Jex

**Affiliations:** Psychology Department, University of Central Florida, Orlando, FL, United States

**Keywords:** appraisal, occupational stress model, stressor, challenge, hindrance

## Abstract

Since its introduction approximately 20 years ago, the Challenge-Hindrance Stress Model (CHM) has been widely accepted both among academic and practitioner audiences. The model posits that workplace stressors can be grouped into two categories. *Hindrance* stressors will interfere with performance or goals, while *challenge* stressors contribute to performance opportunities. These two categories of stressors are theorized to exhibit differential relationships with strain, with hindrance stressors being more consistently linked to psychological, physical, or behavioral strain compared to challenge stressors. Despite the popularity of this model, recent evidence suggests that the proposed differential relationship hypothesis has not consistently held true for all types of strain. Thus, a reexamination or modification of this paradigm is clearly warranted. In the present review, we describe existing evidence surrounding the CHM and describe the rationale for a shifting paradigm. We outline recent advances in research using the CHM, such as novel moderators and mediators, the need to explicitly measure challenge and hindrance appraisal and differentiate between hindrance and threat appraisal, the dynamic nature of these appraisals over time, and the recognition that a single stressor could be appraised simultaneously as both a challenge and a hindrance. Finally, we provide recommendations and future research directions for scholars examining stress and stress management through a CHM lens, including recommendations related to study design, the measurement of stressors, the integration of CHM with other models of stress, and interventions for stress management.

Researchers interested in understanding and decreasing occupational stress depend upon theories and models to ground their investigations of the relationships between stressors and strains (e.g., Sonnentag and Frese, 2003). The Challenge Hindrance Model of Stress (CHM; Cavanaugh et al., 2000), a model that differentiates between types of stressors and their proposed differential relationships with work outcomes, is well-known and widely cited within the occupational stress literature (Mazzola and Disselhorst, 2019). While some researchers offer a more critical view of the support and generalizability of the model (Mazzola and Disselhorst, 2019), others argue for the value of the framework (O’Brien and Beehr, 2019). However, conflicting views do not necessitate that we abandon this framework, but instead highlight an opportunity to challenge and reshape an existing paradigm.

Recent advances in the occupational stress literature offer insight into a more nuanced understanding of the relationships and processes proposed within CHM. In this review, we begin by describing the CHM and its historical roots, review existing evidence surrounding the model, and describe the need for a paradigm shift within the literature. We then describe recent advances in the CHM literature, highlighting studies with conceptual or design features that could facilitate this shift. We conclude with recommendations for future research that builds upon the CHM framework.

## An Overview of the CHM

### CHM Description and Historical Roots

The basic premise of the CHM framework is that stressors can be conceptualized into the two broad categories of *Challenge Stressors* and *Hindrance Stressors*. Challenge stressors are those that may result in strain, but at the same time, are energizing and provide opportunities for feelings of accomplishment, as well as growth and development (Cavanaugh et al., 2000). In most treatments of the CHM, stressors such as workload or impending deadlines are assumed to be challenge stressors. In contrast, hindrance stressors are those that result in strain, but in contrast to challenge stressors, are typically not energizing and do not provide employees with opportunities for growth and development (Cavanaugh et al., 2000). A good exemplar of a hindrance stressor is the concept of organizational constraints (Spector and Jex, 1998), or conditions (e.g., interruptions, poor equipment, etc.) that prevent employees from performing their jobs well.

The CHM is a fairly recent development in occupational stress theory, since it is typically attributed to the article by Cavanaugh et al. (2000) where it was first introduced. Given its recent development, it is easy to overlook its many historical roots. In fact, in recent treatments of this framework (e.g., Mazzola and Disselhorst, 2019) the development of this model was attributed simply to the fact that in many occupational stress studies the signs of correlations were counterintuitive. While this is certainly true, we contend that the historical roots of the CHM go back much further than this.

In fact, the historical roots of this model can be traced back to the widely known Yerkes-Dodson law (Yerkes and Dodson, 1908), which relates one’s level of physiological arousal to task performance. The form of this relationship is an Inverted-U, indicating that there is some optimal mid-range level of arousal that facilitates performance. Although Yerkes and Dodson were not theorizing about the impact of stress on individuals, their model is often invoked to support the idea that the relationship between stressors and many outcomes may deviate from linear, as had often been assumed (e.g., Jex, 1998).

In the organizational literature the Yerkes-Dodson model has been used as a rationale for designing work that has a moderate amount of physiological activation (Scott, 1966), and to explain the link between job complexity and physical health symptoms (Xie and Johns, 1995). In recent years, with the growth of the positive psychology movement, this model has been used as a rationale for arguing that some stressors may actually result in positive outcomes for employees (e.g., Britt and Jex, 2015).

Similar with this notion that strains vary as a function of stressor intensity or duration, Selye (1956) suggested that stressor *type* determined the resulting strain. He coined terms that later become analogous to hindrance stressors and challenge stressors. “Distress” was the term used to refer to stressful situations that exceed individuals’ resources, and “eustress” referred to stressful situations that engage and energize individuals (Selye, 1974).

Another historical foundation of the CHM is the widely known Job Demands-Control (JD-C) model of stress developed by Robert Karasek (Karasek, 1979; Karasek and Theorell, 1990). The most widely known proposition of the JD-C model is that stress is highest when job demands are high and job control (termed “job decision latitude” by Karasek) is low. In essence, what Karasek proposed is that job demands represent physiological challenges to the body and will result in strain when there is no control over how to meet those demands. In more recent years it has been proposed that this interaction occurs only under conditions of low social support.

A less widely cited proposition of the JD-C model (see Kain and Jex, 2010), and one that bears directly on the CHM, is that some jobs are high in demands and at the same time are high in job control (called “Active” jobs). Such jobs are demanding, yet at the same time potentially rewarding, because employees in such jobs have the discretion in how to address those demands. As a result, there is a high probability that individuals holding these types of jobs will accomplish important organizational and occupational goals and reap the benefits of goal accomplishment. While empirical evidence on active jobs is sparse, there is evidence that active jobs can result in positive outcomes (e.g., Van Yperen and Hagedorn, 2003).

Another stress theory which is a forerunner of the CHM (e.g., Cavanaugh et al., 2000) is the transactional theory of stress (Lazarus and Folkman, 1984). At its core, the transactional theory is a theory of two forms of appraisal—*primary* and *secondary*. Primary and secondary appraisals are cognitive judgments of events and situations. Primary appraisal represents an individual’s judgments as to whether or not something is a stressor. For example, an impending work deadline may or may not be perceived as stressful, and there are a number of factors that would impact this judgment such as the importance of the deadline, the work tasks necessary to meet the deadline, and the consequences of not meeting the deadline. If something is in fact perceived as a stressor, the next form of appraisal that comes to the forefront, according to the transactional model, is secondary appraisal. Secondary appraisal, also referred to in the transactional model as coping, represents the way(s) that a person chooses to confront a stressor once it is perceived. Moreover, it is theorized that each appraisal occurs simultaneously and that secondary appraisals can inform primary appraisals, such that the available resources an individual has to cope with a stressor may inform how they appraise said stressor (primary appraisal).

In the previous example, a person with an impending deadline can respond to this stressor in a number of ways. For example, he or she can plan to spend more work time on the deadline, or ask for organizational resources to help meet the deadline—both of which would appear to be functional responses since they would likely increase the probability of actually meeting the deadline. On the other hand, we know that people often cope with deadlines in much less functional ways. They may procrastinate, downplay the importance of the deadline, or even consume alcohol in an effort not to think about the deadline.

The link between the transactional theory of stress and the CHM is rather straightforward since perceptions of challenge stressors and hindrance stressors are appraisals at their core. Interestingly, however, appraisal has not been explicitly incorporated to applications of the CHM until relatively recently and stressors were simply classified by researchers in an *a priori* fashion. This is a point we return to later in the review, but it is a very important one.

### Existing CHM Evidence

Since the model’s publication two decades ago, it has gained traction within the occupational stress literature. Empirical work based on the model continues to grow, and has expanded to incorporate a wider variety of criterion variables (e.g., organizational citizenship and counterproductive work behaviors, Rodell and Judge, 2009; workplace safety, Clarke, 2012), more rigorous methodology (multi-level studies, Idris and Dollard, 2014; daily diary studies, Tadić et al., 2015), and a more nuanced understanding of the boundary conditions and explanatory mechanisms associated with the model (O’Brien and Beehr, 2019).

To quantitatively summarize support for the model across individual studies, multiple meta-analyses have been conducted. For example, LePine et al. (2005) and Podsakoff et al. (2007) categorized stressors studied without an explicit basis in the CHM model as challenge stressors or hindrance stressors, finding partial support for the tenets of the CHM. Specifically, differential relationships were supported for some variables (performance and motivation in LePine et al., 2005 and job attitudes in Podsakoff et al., 2007), but there were also significant positive relationships between both types of stressors and strain in both meta-analyses.

A more recent meta-analysis investigated support for the model among studies using the CHM framework, finding some evidence for CHM predictions when considering task performance as the dependent variable, but not for organizational citizenship behavior, counterproductive work behavior, job attitudes, retention and strain (Mazzola and Disselhorst, 2019). Based on the existing body of evidence, our field remains divided on the utility and generalizability of the model, as evidenced by a recent point-counterpoint piece in which authors recommend to “move away from the current challenge-hindrance model” (Mazzola and Disselhorst, 2019, p. 949) and recommend continued use of the framework to produce “interesting, valuable, and innovative research” (O’Brien and Beehr, 2019, p. 962). It is important to note that these recent point-counterpoint reviews aimed to investigate current levels of support for the model as it currently stands. Although the authors did offer some suggestions to improve the state of CHM literature, their arguments were framed primarily toward the discontinued or continued use of the existing model. The present review, however, expands upon these arguments through an integration of the arguments for and against the CHM, offering conceptual and methodological suggestions to shape future CHM research. In the following sections, we outline the rationale for a shifting paradigm in CHM research and describe advances in the literature that are illustrative of the proposed shift.

### The Need for a Shifting Paradigm

One indicator of the value of any theoretical framework is the extent to which it generates empirical research. If we judge the CHM by this criterion, it has proven to be at least as useful as any occupational stress theory ever developed (see Jex and Yankelevich, 2008 for a description of occupational stress theories). Thus, we are not advocating in this review that the CHM be abandoned or accepted in its current form. Rather, what we are arguing is that evidence has accumulated which suggests that the CHM must undergo major modifications if it is to remain valuable as a guiding framework for occupational stress research. As research and theory-building is an inherently incremental effort, we assert that now is the appropriate time to adapt the CHM based on advances in research design, measurement, and analysis. In this section we briefly summarize the reasons behind the need for a shifting paradigm.

#### Challenge Stressors and Hindrance Stressors Are Fundamentally Appraisals

As we pointed out in the first section of this review, one of the historical roots of the CHM is the transactional theory of stress (Lazarus and Folkman, 1984). Applied to the CHM, this means that stressors are only challenge or hindrance stressors to the extent that they are perceived as such by employees. Unfortunately, this important component of the transactional theory of stress is not commonly incorporated into applications of CHM. Although early work did outline the role of appraisal in the development of CHM hypotheses and attempt to control for variables related to appraisal (Cavanaugh et al., 1998), the CHM did not explicitly incorporate appraisal into how stressors are classified. Instead, stressors are typically universally classified to be either challenge stressors or hindrance stressors.

#### Challenge Stressor and Hindrance Stressor Appraisals Are Not Mutually Exclusive

While the CHM accounts for the fact that challenge and hindrance stressors can exist in the same job (e.g., one can have high workload and perceive organizational constraints), it does not account for the fact that the same stressor can be considered both a challenge and hindrance at the same time (e.g., Webster et al., 2011) and this is problematic for the model. A high workload, for example, can invigorate and provide opportunities for growth. However, it can also prevent employees from spending time with friends or hinder them from meeting other work-related obligations.

#### Challenge Stressor and Hindrance Stressor Perceptions Are Not Static

Since Cavanaugh et al. (2000) proposed the CHM, there has been an explosion of studies in the organizational sciences that have employed within-person data collection (see Beal, 2015 for an excellent discussion of this methodology). While a summary of this research is well beyond the scope of this review, one general conclusion that can be drawn is that perceptions of stressors and strains tend to have considerable within-person variability. That is, people may feel very positive about their work 1 day and much less positive the next day. Given this general finding, we believe that it is entirely possible that an employee could perceive some aspect of their job as a challenge stressors on 1 day yet feel as though these same aspects are hindering on another day.

## Advances in the CHM Framework

Opportunities exist to reshape the CHM paradigm and recent research exemplifies theoretical and empirical advances that can help our field respond to these opportunities. Advances in CHM measurement, study design, and our understanding of the model itself are highlighted in the following sections.

### Advances in CHM Measurement

Since its initial conception by Cavanaugh et al. (2000), research applying this framework made several advances in the way in which the tenets of the model are measured. Specifically, the opportunity to measure appraisals rather than classifying certain stressors as challenge stressor or hindrance stressor *a priori* is discussed. This notion is based on prior research that has found stressors can be appraised as both challenging and hindering (e.g., Webster et al., 2011; Searle and Auton, 2015). Additionally, some researchers argue for the inclusion of a third type of appraisal, threat appraisals.

### Moving From an *a priori* Classifications of Stressors to Appraisal

Stressors were initially classified as either a challenge stressor or hindrance stressor in a universal fashion in the CHM (Cavanaugh et al., 2000) and initial CHM instruments followed this approach. For example, in Rodell and Judge (2009) measure of challenge stressors and hindrance stressors participants are asked to indicate the extent to which they have experienced certain stressors that are considered to be challenge stressors. The items are based on *a priori* classifications, such as workload, responsibility, time pressure, and job complexity. Participants are also asked to indicate whether they have experienced role conflict, role ambiguity, red tape, and hassles at work, which are classified as hindrance stressors in an *a priori* fashion. Although these measures do build upon previous work by measuring a variety of stressors considered to be challenge stressors or hindrance stressors, *a priori* classification of a stressor in this manner can be problematic for both theoretical and empirical reasons.

First, as we previously mentioned, the *a priori* classification of stressors is not consistent with the transactional theory of stress, a foundational theory for the CHM (Cavanaugh et al., 2000). A central tenet of this theory is that individuals make an appraisal of a stressor as a hindrance if they feel like there are obstacles blocking them from achieving their work-related goals (Lazarus, 1991). In contrast, when a stressor offers an opportunity for mastery and growth it should be appraised as a challenge stressor (Lazarus and Folkman, 1984). Although prior research has supported that some stressors have a tendency to be related to positive and negative outcomes (LePine et al., 2005), the value of measuring individual appraisal cannot be overstated.

Second, research has provided evidence that measuring appraisal accounts for unique variance in strain outcomes (Searle and Auton, 2015). This indicates that perceptions of stressors such as workload is meaningful above and beyond the content of the stressor itself, and explaining increased variance bears theoretical importance given that it means findings are better grounded in expected factors as opposed to error. Yet, the current measurement of challenge stressors and hindrance stressors largely ignores the subjective appraisal of stressors. A recent meta-analysis found that challenge stressors and hindrance stressors were not always related to positive and negative outcomes as expected based on the CHM (Mazzola and Disselhorst, 2019). As Mazzola and Disselhorst note, some of these inconsistent findings may be explained by the fact that many of the studies included in the meta-analysis did not measure challenge and hindrance appraisal and relied on *a priori* classification.

Give these findings, we argue that the needed shift in the existing CHM paradigm will incorporate individual appraisal of challenge stressors and hindrance stressors more often rather than relying on *a priori* classifications, and we highlight research that has incorporated this approach. For example, Webster et al. (2011) examined the relationship between two traditionally categorized hindrance stressors (role ambiguity and role conflict) and two challenge stressors (workload and responsibility for things) with challenge and hindrance appraisal. They found that these stressors were significantly related to both challenge and hindrance appraisal, with the exception of responsibility which was only positively related to challenge appraisal. These findings are consistent with the idea that stressors can be simultaneously appraised as both sources of challenge and hindrance simultaneously (Lazarus and Folkman, 1984). More recently, Searle and Auton (2015) also found that stressors have the potential to be appraised simultaneously as challenging and hindering. Specifically, the authors found that time pressure, a stressor typically categorized as a challenge stressor, was appraised as both a challenge and hindrance to a similar extent. The *a priori* classification framework may present some challenges for understanding findings such as these.

Although one of the primary theses of the current review is that a shifted CHM paradigm will incorporate appraisals of stressors as challenge, hindrance, or threat, there may be situations where measuring appraisal may not be of feasible or advisable. Considerations should be made to how appraisals may influence participant response burden or the generalizability of previous findings from archival data and meta-analyses. In regard to research design, there are a number of instances in which appraisals should not be measured. Appraisals should not be measured in retrospect, given that the anticipated positive or negative outcome that influenced their appraisal may have already occurred and distorted their recollection of their initial appraisal. We also assert that there may be little value in asking participants how they generally appraise a stressor or event, given within-person variation in appraisal (Lazarus and Folkman, 1984). Instead, appraisals should be framed to a specific situation (Searle and Auton, 2015).

As the measurement of CHM appraisals is a relatively recent phenomenon (e.g., Webster et al., 2011), more work is needed to identify the situations in which researchers should or should not measure appraisals, as well as to advance the measurement of our objective work environment and our subjective experience of that environment (e.g., Frese and Zapf, 1999; Perrewé and Zellars, 1999). We do not assert that research based on *a priori* classification is invalid, but rather that within a shifted CHM paradigm, researchers will opt for the nuance and precision of explicit measurement of appraisal when possible and when appropriate for the research design. Researchers interested in measuring appraisals are referred to Webster et al. (2011), who measured appraisal by presenting participants with the definitions of challenge stressors and hindrance stressors and asking participants about the degree to which the stressor represented that definition. Readers are also referred to Searle and Auton (2015), who measured appraisals in terms of anticipated future impact (i.e., “will make my work challenging” on an agreement-based Likert scale).

#### Distinguishing Between Hindrance and Threat Appraisals

Some occupational stress researchers have also argued for the value of distinguishing between hindrance and threat appraisal. Incorporating threat appraisal into the CHM is more consistent with the theory (i.e., Lazarus and Folkman, 1984 transactional theory of stress) that the framework is derived from (Tuckey et al., 2015). Over the years, Lazarus and Folkman have suggested that an individual’s appraisal of a stressor may depend on its opportunity for personal gain and mastery (challenge appraisal), potential to inhibit goal attainment (hindrance appraisal), or the possibility that they will lead to loss or harm in the future (threat appraisal). Yet, studies incorporating threat appraisal in the CHM are sparse.

One study that has examined all three types of appraisal was conducted by Tuckey et al. (2015). These authors argued that it is important to distinguish between hindrance and threat appraisal, given that both have negative valence, but differ slightly in their theorized outcomes. Specifically, individuals make threat appraisals in situations that may result in personal harm or loss (Tuckey et al., 2015) and hindrance appraisals are made when goals are obstructed. Tuckey et al. (2015) argue that it is not inherently problematic for the CHM framework that both hindrance stressor appraisals and threat appraisals exist; it is instead problematic that prior studies have assumed and treated hindrance stressors as equivalent to stressors that are appraised as threatening. Indeed, Tuckey et al. (2015) supported a three-factor structure of challenge, hindrance, and threat appraisal, and provided evidence that the three are differentially related to different forms of affect. Challenge appraisals were positively related to positive affect, threat appraisals were positively associated with anxiety and anger, and hindrance appraisal was positively associated with fatigue. Although more research is clearly needed, based on the theoretical underpinnings of the CHM’s basis, we argue that future research should consider the inclusion of threat appraisal in a shifted CHM paradigm. Doing so would allow researchers to test more precise hypotheses about how perceptions influence stressor-strain relationships.

### Advances in CHM Temporal Considerations

As previously mentioned, there is considerable within-person variation in most stressors and strains (Beal, 2015). Additionally, researchers contend that the stress process is dynamic rather than static (McGrath and Beehr, 1990; Rosen et al., 2020). However, little research has sought to examine the temporal aspects underlying peoples’ experience of stress, such as fluctuating appraisals of stressors. Theories based on appraisal (e.g., the transactional theory of stress; Lazarus and Folkman, 1984) highlight the importance of considering the acute event on which appraisals are based, as well as amount of resources available to direct toward stress-inducing stimuli. Given that events and availability of resources will differ over time, there is a great deal of value in accounting for temporal dynamics in stress research.

Based on this logic, not only should the appraisals of stressors differ over time, but the relationships between challenge stressors, hindrance stressors, and strain should also show temporal fluctuations. When an individual is able to predict and plan responses to a stressor, they are better able to invest their energy and efforts toward preparing for the stressor (Lazarus, 1991; Parke et al., 2018; Rosen et al., 2020). As a result of anticipation and planning, they should experience less strain compared to instances when they are not able to foresee a stressor.

Although this notion is currently understudied, a recent study by Rosen et al. (2020) highlights the value of considering the stability of challenge and hindrance stressor appraisals in the shifted CHM paradigm. When challenge stressors were stable from week-to-week, individuals were better able to anticipate stressors, relative to when challenge stressors fluctuated. As a result of anticipating stressors, individuals with stable challenge stressors appraised their stressors as challenging and ultimately experienced less overall stress. Individuals who experienced more fluctuations in challenge stressors exhibited worse task performance and reported greater subjective stress due to lower stressor anticipation and greater hindrance appraisals (Rosen et al., 2020). In sum, these findings provide preliminary evidence that accounting for the dynamic nature of challenge stressors may explain when challenge stressors are beneficial for employees and when they are detrimental.

Our review of the literature found no studies that have drawn from the CHM to examine the variability of hindrance stressors. However, a study conducted by Matta et al. (2017), may provide some evidence to help theorize how the fluctuation of hindrance stressors may result in negative outcomes. Matta et al. (2017) conducted both a lab and field study that examined the effects of consistent and inconsistent fair treatment. Interestingly, in their lab study, the authors found that being treated inconsistently fair and unfair resulted in worse physiological stress than being treated consistently unfair. Additionally, in their follow-up field study they found that people who experienced more justice variability were less satisfied with their job and more emotionally exhausted at the day level.

The theoretical mechanism that explains these findings is posited to be the uncertainty individuals feel when they experience inconsistency at work (Matta et al., 2017). Across a wide variety of contexts, when individuals experience uncertainty they feel less control and ultimately more stress (Bordia et al., 2004; Matta et al., 2017). Applied to hindrance stressors, it is possible that fluctuations in hindrance stressors are worse for well-being relative to experiencing high levels of this type of stressor regularly. For example, if an individual sporadically has to cope with role conflict, they may experience worse strain than an individual who consistently deals with role conflict because they are unable to anticipate the hindrance stressors and mobilize resources to cope with it. However, research has yet to investigate how fluctuations in hindrance stressors between time points impact individuals’ ability to anticipate stressors and mobilize coping resources to deal with their stress.

Overall, accounting for the temporal dynamics of stressors sheds light on whether challenge stressors result in positive or negative outcomes for employees, which has theoretical implications for the CHM (Cavanaugh et al., 2000). Namely, accounting for changes in the amount of challenge stressors employees experience is important to understand when hypothesizing about the relationship between stressors, appraisals, and subsequent outcomes. Such findings also have important practical implications. Based on the idea that challenge stressors should promote goal attainment at work, researchers (e.g., LePine et al., 2005) have recommended that managers and supervisors provide their employees with challenging opportunities. However, if an employee is not able to predict or anticipate when they will be faced with a greater workload or be placed under time pressure, they are likely to experience strain (Parke et al., 2018; Rosen et al., 2020). Therefore, managers and supervisors should be cognizant that challenge stressors have the potential to result in mastery and growth when an employee has the ability to predict and allocate coping resources to that demand. Perhaps building in planned opportunities for challenge into employee training and development plans would be an effective way of achieving this goal.

### Advances in Forms of CHM Relationships

Another potential explanation for the inconsistent support for the tenets of the CHM could lie in the fact that curvilinear relationships have not been explored extensively. Earlier in this review, we noted that the historical roots of CHM acknowledge the possibility of non-linear relationships (Yerkes and Dodson, 1908). Challenge stressors may have the potential for growth and mastery, but when they are experienced in excess, employees may not obtain the positive benefits challenges have to offer. For example, responsibility for tasks at work is commonly characterized as a challenge stressor (Cavanaugh et al., 2000; Rodell and Judge, 2009). However, benefits and goal attainment may only occur when individuals have moderate responsibility for certain aspects of their work. In contrast, when an individual has very little responsibility, they may not be given the opportunity to experience growth. On the other hand, when they experience too much responsibility, they may feel overwhelmed and not perform to their fullest. Therefore, it is possible that when it comes to challenge stressors, an individual may experience too much, or little, of a good thing to reap the benefits of said stressor (Mazzola and Disselhorst, 2019).

The possibility of curvilinear relationships was explored in Cavanaugh et al. (1998) working paper, but much of the CHM literature seems to default to linear hypotheses. Although understudied, there is some evidence that challenge stressors have the potential to have curvilinear relationships with outcomes. For instance, Baer and Oldham (2006) found that time pressure had a curvilinear relationship (inverted U-shape) with creativity for employees who reported high levels of openness to experience and worked in an organization that reported high levels for creativity. Although not the primary focus of their study, Rosen et al. (2020) conducted a polynomial regression analysis and found that greater fluctuations in challenge stressors from week-to-week had a curved response surface. This finding suggests that as the difference between past challenge stressors and current stressors becomes greater, individuals are increasingly less attentive and able to anticipate stressors at work. In regard to stress appraisal, the curved estimate for the difference between past and current challenge stressors, was significant for both challenge and hindrance appraisal. In other words, as fluctuations between past and current stressors increase, individuals make even fewer challenge appraisals and increasingly more hindrance appraisals. Increased consideration of non-linear forms of CHM relationships, particularly when integrated with appraisal-based measurement rather than *a priori* classification, will foster a more nuanced understanding of the complexities of stressor-strain relationships in a shifted CHM paradigm.

### Advances in the CHM Itself

While some recent CHM advances relate to the measures and analytic techniques used in studies examining the model, other advances relate to the CHM itself. That is, recent research on boundary conditions and explanatory mechanisms provide a greater understanding of how CHM components related to one another and the conditions under which CHM tenets are supported.

#### Greater Understanding of Boundary Conditions

The examination of moderator variables in the CHM (Cavanaugh et al., 2000) is a promising avenue of research for two primary reasons. First, research may provide evidence that certain traits, states, or environments promote challenge and hindrance appraisals. Second, if these traits, states, and environments are found to benefit individuals in how they appraise stressors, there may be opportunities for interventions in the workplace that bolster such conditions. We refer readers to a discussion of CHM boundary condition research by O’Brien and Beehr (2019), who describe individual difference (e.g., conscientiousness; Abbas and Raja, 2019), psychological states (e.g., recovery experiences; Bennett et al., 2018), and employee actions (e.g., proactive coping; Searle and Lee, 2015) that have been found to moderate the stressor-strain relationship in a CHM framework. They also highlight variables, such as social support, which have yet to be investigated in a CHM framework and may present a fruitful avenue in future research.

For future CHM boundary condition research, one individual difference that has been suggested (e.g., Mazzola and Disselhorst, 2019) to impact how individuals appraise stressors is stress mindset. Stress mindset is defined as an individual’s belief that stress has the opportunity to promote positive outcomes, such as productivity and well-being (stress-is-enhancing), or that stress is detrimental for such outcomes (stress-is-debilitating; Crum et al., 2013). In the context of the CHM, individuals who have a stress-is-enhancing mindset may be more likely to appraise stressful events as challenges rather than a hindrance or threat. In contrast, individuals who have a mindset that stress is debilitating may appraise stressful events as more of a threat or hindrance, rather than a challenge.

A similar individual difference that may function as a moderator between stressors and appraisal is coping self-efficacy. Coping self-efficacy has been defined as an individual’s confidence in their ability to effectively cope with stress (Chesney et al., 2006). Alluding to the transactional theory of stress (Lazarus and Folkman, 1984), an individual’s primary appraisal of a stressful event will be impacted by their perception of coping resources. Therefore, when an individual with high coping self-efficacy is confronted with a stressful event, they may be inclined to appraise the event as more challenging, rather than threatening or hindering, relative to a person with low coping self-efficacy. Alternatively, it is possible that the appraisal process is not impacted, rather the relationship between different types of appraisals and outcomes, will be less severe for individuals who feel they can effectively cope with stress.

Another individual difference that may warrant future research as moderator is goal orientation. Previous studies have conceptualized workload, time pressure, and responsibility as challenge stressors in the CHM (Cavanaugh et al., 2000; LePine et al., 2005; Rodell and Judge, 2009) because they present opportunities for personal growth and mastery. However, constructs such as these may only result in greater task performance and growth, through challenge appraisal, depending on an individual’s goal orientation. Research on goal orientation has identified two types of goal orientations: performance and learning (Button et al., 1996). Performance goal orientation is described as a motivation to avoid negative judgments related to one’s competence and attain favorable judgments from personal accomplishments and performance. Learning goal orientation is characterized as a motivation to understand new concepts and becoming competent in certain activities (Button et al., 1996).

Applied to the CHM when individuals with high performance goal orientation are faced with a high workload, they may appraise this situation as a challenge and perform better than individuals with low performance orientation. Similarly, when individuals with high learning goal orientation are given responsibility over things at work, they may be motivated to develop their competence and as such experience greater mastery over those responsibilities. Under a shifting CHM paradigm, researchers are encouraged to continue to search for moderators that may help us understand *when* and *for whom* CHM tenets are supported, while keeping in mind criteria for useful research on boundary conditions (Murphy and Russell, 2017).

#### Greater Understanding of Explanatory Mechanisms

Research examining CHM mediators is important because it sheds light on the theoretical mechanisms that explain the relationships between challenge stressors, hindrance stressors, and outcomes. A key theme in this review has been the importance of appraisal in CHM, which raises the question as to whether appraisal mediates CHM relationships. O’Brien and Beehr (2019) note that when appraisals are included in CHM studies, mediational hypotheses are generally supported (although there may be more evidence for appraisal as a mediator of the hindrance stressor-strain relationship than the challenge stressor-strain relationship; Gerich, 2017). In addition to appraisal, several other variables have been investigated as mediators in the CHM framework.

Recently, work engagement has been examined as a mediating variable in the context of organizational change. Kaltiainen et al. (2019) examined how individuals’ appraisal of a merger changed over time through latent change score modeling. They found that individuals who initially perceived the merger as threatening were less engaged at work during the merger and experienced a significant increase in threat appraisal as the merger continued to be implemented. This study is influential for the CHM because it examines how appraisals of organizational change may fluctuate over time and provides evidence that this may be due to decreased engagement at work.

Another novel mediator that has been examined is stressor anticipation. Evidence has supported that when traditionally measured challenge stressors fluctuate, individuals are less able to anticipate stressors and, as a result, make more hindrance appraisals and less challenge appraisals (Rosen et al., 2020). We contend that future research should investigate anticipation as a mediating mechanism between stressors and appraisal given recent literature that has shown stressors (Rosen et al., 2020) and stress appraisals (Kaltiainen et al., 2019) fluctuate over time. Within a shifted CHM paradigm, we encourage researchers to continue explorations into *how* and *why* CHM tenets are supported.

## Recommendations and Future Directions

Throughout this review, we have argued for the need for a shifted CHM paradigm as opposed to complete acceptance of the model in its current form or complete abandonment of the model. We believe that in addition to attention to some of the novel and innovative research advances highlighted in this article, adhering to several recommendations will foster a needed paradigm shift and will add further value to an influential model.

### Recommendations for Stress Models and Theories

The CHM provides a useful lens for understanding stressor-strain relationships. However, with the exception of appraisal-based theories of stress, occupational stress theories have rarely incorporated challenge and hindrance distinction into their propositions. We discuss opportunities for integration with other influential work stress theories and hope that more authors will consider integration of CHM with these theories when appropriate for the aims and scope of their research.

#### Job Demands-Resources Theory

As discussed previously, the Job Demands-Control Model (JD-C; Karasek, 1979) can be considered as part of the historical roots of the CHM, given that a job with high demands and high control could represent the presence of challenge stressors. Thus, this model and advancements of this model such as the Job Demands-Resources Model (JD-R; Demerouti et al., 2001; Bakker and Demerouti, 2017) represent prime opportunities for integration with CHM. However, studies using a JD-R framework do not typically consider whether demands are appraised as a challenge or hindrance stressor based on available resources to address the demand. Additionally, the tenet described above (i.e., high demands and high control or resources) is not cited as often as the tenet relating to high demands and low control or resources, which can lead to an interpretation of all job demands (i.e., stressors) as strain-inducing. In other words, based on the most often-cited propositions of JD-R, some researchers could interpret the “demands are bad” logic of JD-R and the “some stressors are good” logic of CHM as incompatible. These theories could be further integrated with a greater consideration of the “active jobs” component of JD-C in which positive outcomes are observed even in the presence of high demands and by incorporating appraisals of demands as challenge or hindrance stressors as mediators in JD-R models.

#### Conservation of Resources Theory

Conservation of Resources (COR) theory proposes that stress is the result of situations and conditions that lead to resource loss or are anticipated to lead to resource loss (Hobfoll, 1989). The theory additionally posits that people are motivated to preserve current resources and acquire more resources. These core propositions can be reframed in light of findings and theory related to the CHM. Applied to COR theory, hindrance stressors can be understood as circumstances that primarily threaten current resources or hamper opportunities to acquire more resources. On the other hand, challenge stressors could be understood as “risky investment” circumstances. These are circumstances where there is opportunity for resource gain (e.g., learning) yet also the possibility for loss of invested resources (e.g., time). Consequently, research on CHM may draw from COR theory to formulate hypotheses and research questions. In their working paper on challenge and hindrance stress, Cavanaugh et al. (1998) draw on COR theory when framing challenge stress as associated with anticipated gains, highlighting the synergies between these two frameworks.

#### Vitamin Model

The Vitamin Model’s (Warr, 1987) propositions deal with the concepts of diminishing returns and the “too-much-of-a-good-thing” principle. First, some job resources theorized to alleviate strain or promote well-being (e.g., salary; safety) will have a positive effect up until a certain point, after which further increases in the resource will no longer have any effect. Other job characteristics (e.g., job autonomy) will have inverted u-shaped effects, such that especially low or high levels of the resource will have a negative effect on well-being. In other words, stressors and resources each have a “sweet spot” at which they exhibit the strongest relations with strain.

This model’s tenets are not directly analogous to the CHM, but the models may be compatible. Curvilinear relations among challenge stressors, hindrance stressors, and strain may emerge in two ways. First, characteristics of a stressor may have curvilinear relations in how well they predict appraisals. For some individuals, situations that require moderate levels of social interaction may be appraised as a challenge while high levels of social interaction may be appraised as threatening. Second, appraisals themselves may have curvilinear relations with strains. There may be specific levels of challenge appraisal that are perceived as tolerable and even desirable. After a specific point, however, challenge appraisals may still predict emotional exhaustion. This logic highlights the importance of testing curvilinear CHM relationships, and within stressor-strain relationships more generally (Karanika-Murray, 2010).

### Recommendations for Study Design and Measurement

We encourage researchers to adhere to the measurement and design-related recommendations made throughout this review, including direct measurement of appraisal of stressors as challenging or hindering when appropriate, a consideration of the differentiation between hindrance appraisals and threat appraisals, greater attention to temporal influences in appraisals and the relationships between stressors and strain, attention to the possibility of non-linear relationships, and continued exploration of moderators and mediators pertinent to the CHM. Although each recommendation may not be relevant or feasible for all future CHM studies, attention to such recommendations when possible will better acknowledge the foundational theoretical models on which CHM is built, better acknowledge the complexities of occupational stress research, and may help address inconsistent findings in CHM research. In addition to the recommendations offered throughout this review, we encourage researchers to consider the following recommendations for future CHM research design.

#### Understanding the Content of Appraisals

Despite the numerous advantages of a quantitative approach, there have been recent calls for greater use of mixed methods research designs in occupational health psychology research (Schonfeld and Mazzola, 2013). Such designs combine the advantages of qualitative and quantitative approaches, allowing researchers to gather rich accounts of context while summarizing differences and relationships with parsimony. Applied to CHM, there is still much to be learned about the context in which challenge stressors or hindrance stressors occur. An end goal is inherent in the basic tenets of CHM. A challenge stressor provides growth and development, presumably for some imagined end point (i.e., a promotion, a chance to implement a skill in a new context). A hindrance stressor interferes with an employee’s work life, and we can infer that stressors thwart the attainment of some goal (i.e., completing job tasks to the best of their ability, spending time with friends or family). Lastly, a threat represents a possible personal harm or loss related to a goal (e.g., maintaining physical health, financial security). Applying a mixed methodology approach would allow researchers to gather more detailed information about the stressor, the end goal that is either being facilitated or hindered, or the anticipated loss or harm. In essence, we will be able to understand *why* a stressor is challenging, hindering, or threatening rather than merely measuring the presence or appraisal of the stressor.

Moreover, existing conceptualizations of stressors as either challenge or hindrance stressors excludes work events that may be stressful but are not traditionally conceptualized as a stressor. For example, an upcoming performance review, an interruption at work, an email, or a request for help are all events that could vary in the degree in which they are appraised as a challenge or hindrance. That is, a performance review may be appraised differently if an employee has reason to believe they will be promoted based on their performance this year or reprimanded if they have performed poorly. However, the current conceptualization of stressors as either a challenge or hindrance does not include such events, nor does it provide clear propositions about how such work events should be conceptualized as either a challenge or hindrance. Gathering rich event-related information would help elicit a greater understanding of additional stimuli that may be appraised as a challenge, hindrance, or threat beyond those stressors already included in *a priori* classifications.

#### Expanding the Levels of Analysis

The study of occupational stress has primarily focused on individual-level predictors and indicators of strain (Bliese and Jex, 1999). This is also true of research examining stressor-strain relations through the CHM lens (Cavanaugh et al., 2000). Research using individuals as the primary unit of analysis is appropriate for identifying general trends in stressor-strain relationship and the impact that individual-level factors (e.g., personality, attitudes) have in these relations. They are also appropriate for examining inter-individual differences in perceptions of shared experiences (e.g., company layoffs, policy changes). However, findings can be extended by studying the CHM at both lower-order levels of analysis (i.e., within-person, daily experiences) and higher-order levels of analysis (i.e., team-, department-, or organization-level predictors and outcomes). Shifting focus to different levels of analysis will allow researchers to expand upon basic lines of inquiry in stress research.

##### Within-Person Design Considerations

Within-person research designs involve collecting observations from individuals at multiple time points to capture intra-individual variation in variables of interest. Several research questions related the CHM necessitate the use of within-person designs. Appraisals, by definition, are context-dependent cognitive judgments of specific circumstances or situations. For the vast majority of research questions, it would be inappropriate to ask participants to appraise general stressors (e.g., interpersonal conflict) because the extent to which stressors are judged to be challenging or hindering will vary depending on the circumstances of the particular situation (Searle and Auton, 2015). Research questions that aim to understand the factors that predict appraisals, how appraisals change over time, or the outcomes of appraising specific events, are therefore an excellent fit for experience sampling method (ESM) and longitudinal designs.

##### Higher-Level Design Considerations

The study of challenge and hindrance appraisals has been primarily studied at the individual level and at the intra-individual level (an exception is Pearsall et al., 2009). This leaves many opportunities to better understand how the challenge-hindrance distinction affects occupational health dynamics at higher levels of analysis (i.e., teams, departments, and organizations). Numerous research questions can only be studied at this level of analysis. For example, researchers may investigate whether certain team-level variables (e.g., task interdependence, team resilience, norms) make it more likely for employees to appraise events as challenges or hindrances, the extent to which challenge and hindrance stressors influence team processes and outcomes, the outcomes of high or low variability in appraisals of a shared experience.

Research at higher levels of analysis may be conducted using both field studies and laboratory studies. Design choice will be dictated to some extent by the research question and inferences that a researcher wants to make. Research questions that are concerned primarily with variables that occur naturally over time or occur meaningfully at the organizational level of analysis will be a better fit for field research. Research questions that involve specific team inputs that can be experimentally manipulated (e.g., tasks) will be strong choices for laboratory designs.

#### Experimental and Quasi-Experimental Design Considerations

Despite the stronger case for casual inferences that can be made based on experimental and quasi-experimental designs, they are underutilized in occupational stress research (Chen et al., 2013). Applied to the CHM, most of the literature would not support that hindrance stressors *cause* strain and that challenge stressors *cause* positive outcomes, only that these variables are *related* (and temporally aligned with expectations in the case of longitudinal research). Inclusion of experimental studies with random assignment, as well as quasi-experimental studies with strong design features that mitigate threats to validity, add to the cumulative body of evidence in a way that better supports casual inferences (Shadish et al., 2002). We encourage researchers employing a CHM lens to consider the totality of study designs in their researcher toolkit, such as vignette studies that experimentally manipulate a variable of interest (i.e., the presence of absence of challenge or hindrance stressors) and intervention studies that experimentally manipulate the presence of an intervention based on CHM.

### Recommendations for Interventions

Despite the simplicity and popularity of the model, the CHM has yet to be fully explored from an intervention perspective. In a recent systematic review of occupational health psychology interventions, none of the coded studies cited CHM as a basis for the intervention being evaluated (Burgess et al., 2019). Interventions based off of the CHM framework could adopt several approaches. First, employees could be trained on the potential value of appreciating challenges, combatting any pervasive avoidance response to all stressors. Prior to an intervention based on this educational approach, an employee could universally avoid or react poorly to all stressors, even challenge stressors. Through the intervention they would reshape appraisals and behaviors toward challenge stressors. Such logic is consistent with the approach of providing psychoeducation on eustress and distress in a stress management intervention (Le Fevre et al., 2006).

Second, a job crafting perspective could encourage employees to seek out more features of their job that are challenging and fewer that are hindering. Job crafting, thought of as “individual job redesign” (Tims and Bakker, 2010, p. 1), refers to employees actively changing the boundaries, conditions, relationships, and meaning involved in their work (Wrzesniewski and Dutton, 2001). In fact, the Job Crafting Scale (Tims et al., 2012) represents an integration of the CHM with the JD-R Model, as the items reference increasing challenging job demands, decreasing hindering job demands, and securing social and structural resources. Although not explicitly based on CHM, job crafting interventions (particularly those in which protocols mirror the factors of the Job Crafting Scale), could be seen as interventions that are related to CHM. Although the body of evidence on job crafting interventions is still growing, preliminary support exists for their utility in supporting employee well-being (Demerouti et al., 2019).

Finally, a job design perspective would suggest that interventions based on CHM should design work to decrease hindrances and build in more challenges. Whereas the job crafting approach would depend on proactive redesign efforts from the part of the employee, a job design perspective would involve an organization creating or restructuring a job in a way that better supports motivation and well-being (e.g., Hackman and Oldham, 1976). From a CHM perspective, the goal of a job redesign effort would be to minimize hindrance stressors and maximize challenge stressors.

Strategies used in job design or redesign efforts include job rotation (rotating jobs to promote flexibility, awareness, and motivation), job enlargement (expanding the breadth of work tasks and responsibilities), and job enrichment (increasing autonomy in the manner of fulfillment of job tasks; Belias and Sklikas, 2013). Although these strategies are expected to lead to enhanced satisfaction and motivation, they could also introduce some new stress into an employee’s work life due increased responsibility and expanded tasks. Yet, through a CHM framework these new stressors would be more likely to be appraised as challenge stressors and support positive outcomes. As the literature on interventions to improve employee quality of work life continues to grow and evolve, researchers and practitioners may wish to further investigate the utility of a CHM perspective.

## Conclusion

Despite the relatively recent introduction of the CHM (Cavanaugh et al., 2000), the occupational stress literature has responded with frequent adoption and consideration of the model (Jex and Yankelevich, 2008). However, recent literature argues both for and against the continued use of the model as it currently stands (Mazzola and Disselhorst, 2019; O’Brien and Beehr, 2019, respectively). We argue that a shifted paradigm that better accounts for challenge and hindrance stressors as appraisals, the possibility of the coexistence of challenge and hindrance appraisals for a single stressor, and for temporal dynamics is needed to advance CHM and enhance its value to the occupational stress literature.

We highlight advances in CHM research as exemplars of this shifted paradigm, including studies that further the measurement of CHM appraisals, studies that explore temporal dynamics and non-linear forms of relationships, and studies that extend our knowledge of how and when CHM predictions hold true through examinations of moderators and mediators. We also offer recommendations for researchers intending to respond to this call for a shifted CHM paradigm, including a greater understanding of context, expansion of levels of analysis, and greater attention to CHM in workplace stress interventions. We believe that a shifted paradigm, informed by these advances and recommendations, will address shortcomings of the CHM model and preserve the utility of the influential occupational stress framework.

## Author Contributions

KH organized literature review and writing efforts and was primarily responsible for summarizing previous CHM research and the intervention section. WN was primarily responsible for researching and writing the recent advances section. MD was primarily responsible for researching and writing the future recommendations section. SJ was primarily responsible for writing the historical roots and need for shifting paradigm sections. All authors played an equal role in manuscript editing and made a substantial contribution to this manuscript.

## Conflict of Interest

The authors declare that the research was conducted in the absence of any commercial or financial relationships that could be construed as a potential conflict of interest.
